# Identification of Poor-outcome Biliopancreatic Carcinoma Patients With Two-marker Signature Based on ATF6α and p-p38 “STARD Compliant”

**DOI:** 10.1097/MD.0000000000001972

**Published:** 2015-11-13

**Authors:** J. Martinez-Useros, T. Georgiev-Hristov, A. Borrero-Palacios, M.J. Fernandez-Aceñero, M. Rodríguez-Remírez, L. del Puerto-Nevado, A. Cebrian, M.T. Gomez del Pulgar, A. Cazorla, R. Vega-Bravo, N. Perez, A. Celdran, J. Garcia-Foncillas

**Affiliations:** From the Translational Oncology Division, OncoHealth Institute, University Hospital Fundacion Jimenez Diaz (JM-U, AB-P, MR-R, L.P-N, AC, MTGP, JG-F); Hepatobiliary and Pancreatic Surgery Unit, General and Digestive Tract Surgery Department, University Hospital Fundacion Jimenez Diaz (TG-H, AC); Department of Pathology, University Hospital Clinico San Carlos (MJF-A); and Department of Pathology, University Hospital Fundacion Jimenez Diaz, Madrid, Spain (AC, RV-B, NP).

## Abstract

Biliopancreatic cancer is one of the most aggressive solid neoplasms, and incidence is rising worldwide. It is known that ATF6α is one of the transmembrane proteins that acts crucially in endoplasmic reticulum stress response, and knockdown induces apoptosis of pancreatic cells. Apart from this, p-p38 has been previously correlated with better outcome in pancreatic cancer. Interestingly, ATF6α knockdown pancreatic cells showed increased p-p38. The aim of this study was to evaluate the expression of these 2 proteins, p-p38 and ATF6α, and their correlation with the outcome of biliopancreatic adenocarcinoma patients.

Samples from patients with biliopancreatic adenocarcinoma that underwent pancreaticoduodenectomy from 2007 to 2013 were used to construct a tissue microarray to evaluate p-p38 and ATF6α proteins by immunohistochemistry.

We observed that both markers showed a tendency to impact in the time to recurrence; then a combination of these 2 proteins was analyzed. Combination of ATF6α^high^ and p-p38^low^ was strongly associated with a higher risk of recurrence (hazard ratio 2.918, *P* = 0.013). This 2-protein model remained significant after multivariate adjustment.

We proposed a 2-protein signature based on ATF6α^high^ and p-p38^low^ as a potential biomarker of risk of recurrence in resected biliopancreatic adenocarcinoma patients.

## INTRODUCTION

Biliopancreatic cancer is the fourth leading cause of cancer death in both sexes in the USA. It is estimated that 46,420 new cases of this cancer are diagnosed in 2014—23,530 in men and 22,890 in women. The estimated death number in USA in 2014 was 39,590 cases,^1^ and 227,000 deaths per year worldwide.^2^ Furthermore, statistical analysis from 2001 to 2010 indicates that death rates are rising.^1^

In this kind of cancer, survival can be improved when tumors are detected at an early stage. It has been reported that 5-year survival rate is of 50% when tumors are <2 cm^3^ and close to 100% for tumors <1 cm.^4^ Although these data are encouraging, biliopancreatic cancer is usually asymptomatic, and disease only becomes apparent after the tumor invades surrounding tissues or metastasizes to distant organs.^5^

Up to date, the unique prognostic biomarker approved by the US Food and Drug Administration (FDA) for resectable biliopancreatic cancer is the preoperative levels of carbohydrate antigen 19-9 (CA19-9).^6^ This marker shows a relatively high sensitivity and specificity for this cancer,^7^ which is superior compared with other markers, such as carcinoembryonic antigen (CEA), carbohydrate antigen 50 (CA-50), and pancreatic cancer-associated antigen 2.^8,9^ However, the applicability of this marker is not obvious since other clinical events such as biliary obstruction can increase CA19-9 serum levels,^10^ and up to 10% of the population cannot synthesize CA19-9.^11^ Nevertheless, CA19-9 is considered the best serum marker for biliopancreatic cancer.^12^

To date, surgical resection remains the best option to manage with biliopancreatic cancer in surgically amenable tumors, and survival can be predicted based on pathological characteristics like tumor size, grade of differentiation, and lymph node status.^13^ However, after surgery, there is a lack of validated prognostic or predictive markers to be used in the patient management.^14^ In this sense, there have been several reports on some prognostic molecular biomarkers, such as mothers against decapentaplegic homolog 4 (Smad4) or mucin 1 (MUC1), and also predictive markers including secreted protein acidic and rich in cysteine (SPARC), human antigen R (HuR), or members of the breast cancer type 2 (BRCA2) family.^15–17^

Very recently, it has been reported that high phospho-p38 (p-p38) levels are correlated with a significant overall survival advantage in biliopancreatic cancer. In fact, inhibition of p38 increased growth of pancreatic tumour cells lines in vitro, suggesting that p-p38 expression constrained cell growth through negative regulation of cell cycle at the G1/S and G2/M transitions.^18,19^ Interestingly, p-p38 was elevated in ATF6α knockdown pancreatic cells. Indeed, ATF6α knockdown resulted in an apoptotic phenotype given by a p38 phosphorylation.^20^ The same authors suggested that p38 is an important contributor to nuclear translocation and transcriptional activation of ATF6α.

ATF6α is one of the transmembrane proteins needed to induce response to endoplasmic reticulum (ER) stress caused by hypoglycemia, hypoxia, or accumulation of unfolded proteins during protein synthesis.^21^ Actually, ER stress is critical for pancreatic cells dysfunction and death.^22,23^ ATF6 has 2 isoforms—α and β, and it has been described that double ATF6α and ATF6β- knockout mice die in the embryonary period,^24,25^ which indicates that the presence of at least 1 isoform is essential for survival.

ATF6α is activated in Golgi apparatus,^22,23^ migrates to the nucleus, and stimulates transcription of survival genes to neutralize ER stress avoiding apoptosis and promoting cell survival.^24,25^ Apart from this, ATF6α is expressed in nonstressed pancreatic cancer cell lines and primary rodent islets, and apoptosis is induced after ATF6α knockdown.^26^ Independently of ER stress, ATF6α is considered an important component in the vascular endothelial growth factor (VEGF)-induced vascularization, inducing survival and angiogenesis.^27^ Moreover, high expression of ATF6α has been implicated in the pathogenesis of high-grade hepatocellular carcinomas^28–30^ and chemotherapy resistance.^31,32^

The goal of this study was to analyze the expression of these 2 proteins—p-p38 and ATF6α—with outcome of biliopancreatic adenocarcinoma patients.

## METHODS

### Patient Samples

A total of 53 patients with biliopancreatic adenocarcinoma who underwent pancreaticoduonenectomy from 2007 to 2013 at the Hepatobiliary and Pancreatic Surgery Unit, General and Digestive Tract Surgery Department, University Hospital Fundación Jiménez Díaz, were assessed for eligibility. For this study, 8 patients were excluded because samples had not enough quality to perform immunohistochemistry (n = 4), and patients presented loss of follow-up (n = 3) or duodenum origin (n = 1). Half of the tumors (51%) were originated in the pancreas and the rest were originated in the intrapancreatic bile duct (27%) or in the ampulla (22%). Gemcitabine was used alone or in combination with radiotherapy and 5-fluorouracil (5FU) as adjuvant treatment in 40% of cases. Histopathological grading of the tumors was based on the recommendations by the College of American Pathologists.^33^ A 2-tiered system has been used to grade tumors in 2 groups: low grade was defined as greater than or equal to 50% of gland formation in the tumor and high grade as less than 50% gland formation.

Ethics committee of clinical research of Fundacion Jimenez Diaz Hospital (CEIC-FJD) has evaluated this study and approved it on December 9, 2014, by the act number 17/14. CEIC-FJD also certified this study belongs to RNA-Reg Consolider-Ingenio CSD2009–0080. All patients gave written informed consent for the use of their biological samples for research purposes.

### Tissue Microarray

Samples from 45 patients were used to construct a paraffin block containing 90 cores (2 cores per patient) to allow immunohistochemistry analysis. A hollow needle was used to obtain a tissue core of 0.6 mm in diameter from selected tumor regions in formalin-fixed paraffin-embedded (FFPE) tissues. These tissue cores were then inserted in a recipient paraffin block in a precisely spaced resembling an array pattern. Sections from this FFPE block were cut in a microtome and mounted on a microscope slide to be analyzed by immunohistochemistry.

### Immunohistochemistry and Quantification

Immunohistochemical staining was conducted in 2-μm FFPE tumour sections. Slides were deparaffinized by incubation at 60°C. Biopsies were cut and incubated with PT-Link (Dako) for 20 minutes at 95°C in a high pH-buffered solution. To block endogenous peroxidase holders were incubated with peroxidase blocking reagent (Dako). Biopsies were stained for 20 minutes with a 1:750 dilution of ATF6α antibody (AP08853PU-N, Acris Antibodies) and 1:150 of p-p38 (ab38238, Abcam), followed by incubation with the appropriate anti-Ig horseradish peroxidase-conjugated polymer (EnVision, Dako) to detect antigen-antibody. Sections were then visualized with 3,3’-diaminobenzidine as a chromogen for 5 minutes and counterstained with hematoxylin. Immunoreactivity was quantified as the percentage of positively stained cells over total tumor cells. Quantification for each patient was calculated with the average of both cores by 2 independent pathologists.

### Statistical Analysis

The association of ATF6α or p-p38 expression their combination with time to recurrence after resection and overall survival was assessed. Time to recurrence was defined as the interval between the dates of surgery and recurrence (local or distant). Overall survival was defined as the interval between the dates of surgery and death from any cause. Survival curves were estimated using the Kaplan–Meier method, and significant survival differences between groups were determined by the log-rank test. Univariate and multivariate Cox proportional-hazard models were used to assess the hazard ratios (HRs) and confidence intervals (CIs) of both molecular and clinical variables. In the multivariate analysis, only those variables that were statistically significant in the univariate analysis were included. *P* values ≤0.05 were considered significant. All statistics were performed with the IBM SPSS statistics 20.0.

## RESULTS

### Patient Characteristics

The clinical features of the resected biliopancreatic cancer patients are summarized in Table 1. The sex distribution in our cohort was 40% of men and 60% of women. The median age for this cohort of patients was 66 years (range 37–82 y).

**TABLE 1:**
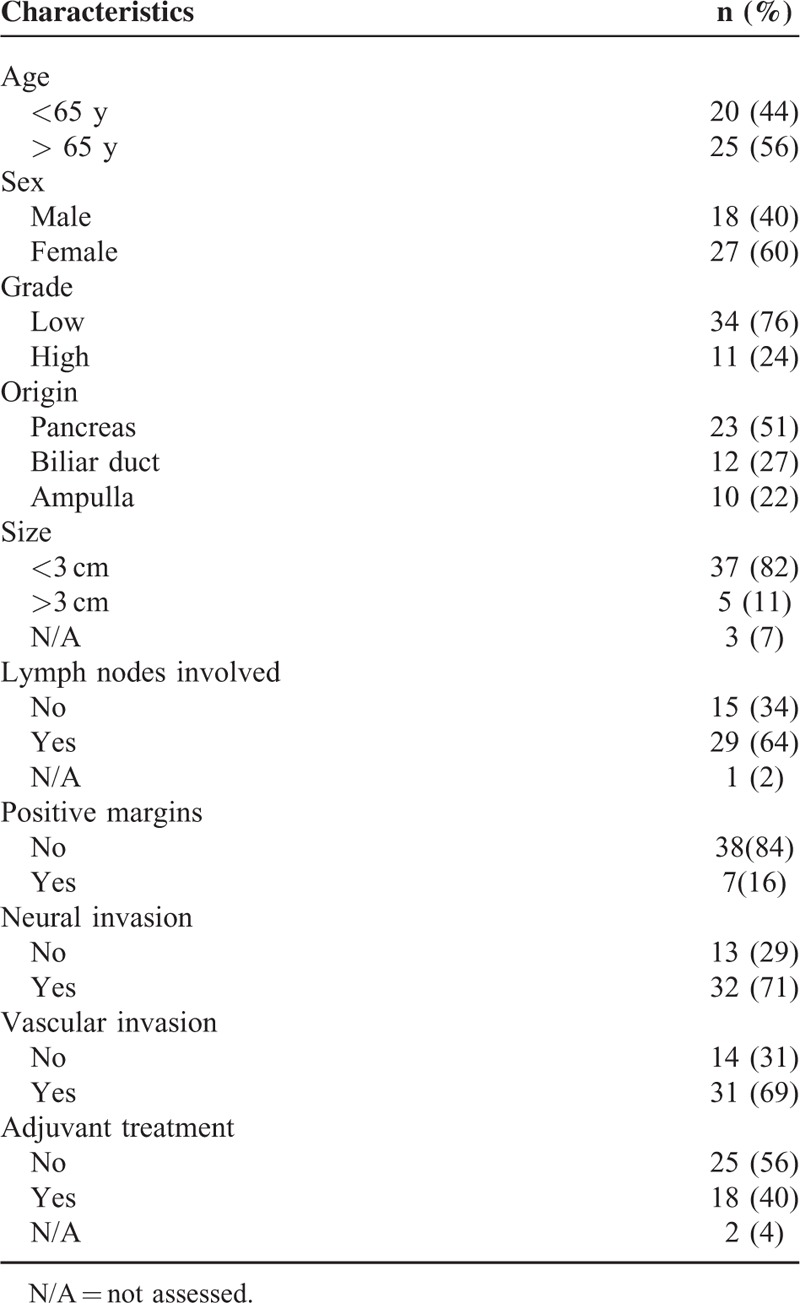
Clinical Characteristics of Biliopancreatic Adenocarcinoma

Most of the tumors were low grade (76%). Metastasis appeared in lymph nodes in 64% of patients; in addition most of the patients had neural and vascular invasion (71% and 69%, respectively). Survival analysis according to tumor origin did not reveal any statistical difference between pancreas, bile duct, or ampulla localization for both time to progression after surgery or overall survival (*P* = 0.956 and *P* = 0.892, respectively, data not shown).

### Combination of High ATF6α and Low p-p38 Levels is Associated with Poor Prognosis in Resected Biliopancreatic Cancer Patients

**FIGURE 1:**
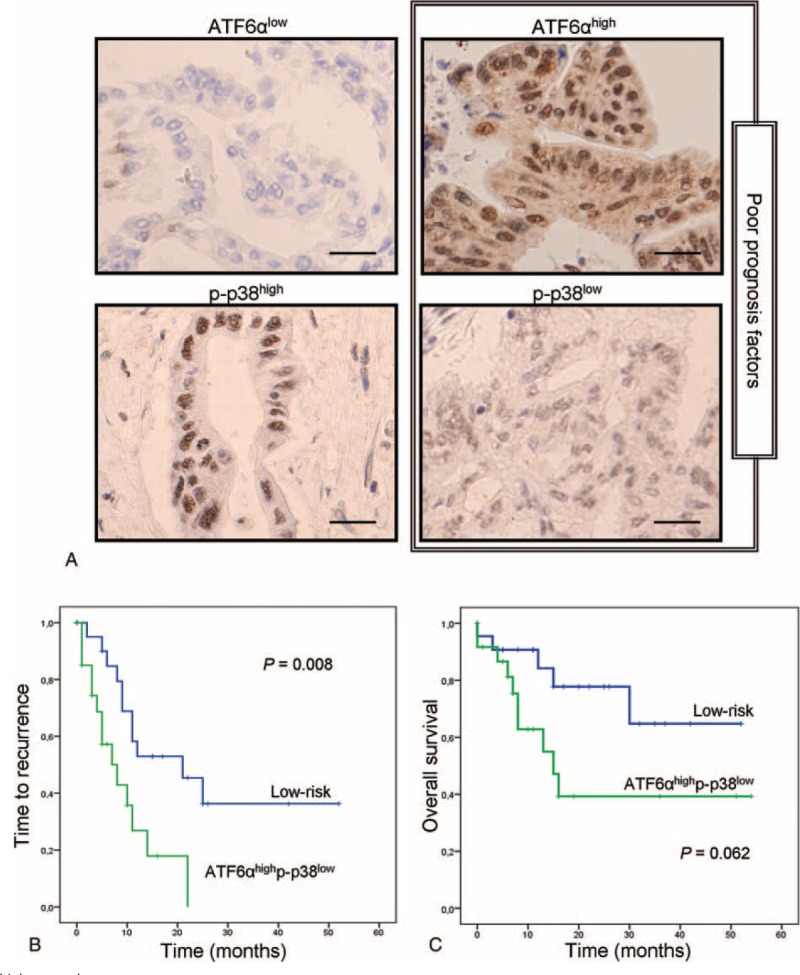
ATF6α^high^p-p38^low^ signature predicts shorter time to recurrence after surgery. A, Four representative immunostaining of ATF6α and p-p38 showing differential expression pattern. B, Kaplan–Meier analysis for time to recurrence after surgery of patients showing ATF6α^high^p-p38^low^ expression (green line) versus low-risk patients (blue line). C, Kaplan–Meier analysis for overall survival of patients with ATF6α^high^p-p38^low^ (green line) versus low-risk patients (blue line).

ATF6α staining had not only basically nuclear localization but also was diffusely detected in the cytoplasm of tumor cells (Fig. 1A). In the cases with high expression, ATF6α was also detected in the nucleus of some stromal cells, although most of the cases showed stronger staining in tumor cells than in stroma. The correlation of ATF6α with outcome of the patients was assessed. For this, patients were stratified into tertiles and the first tertile was established as the cut-off point. Patients with high expression of ATF6α (ATF6α^high^) showed a trend to decreased time to recurrence and overall survival (*P* = 0.1 and *P* = 0.07, respectively) (data not shown).

Expression of p-p38 was seen preferentially in tumor cells with a clear nuclear localization, and also it was detected in isolated fibroblasts (Fig. 1A). In this case, patients were stratified in low or high-expression groups using the median as cut-off point. Patients with low p-p38 (p-p38^low^) levels showed a trend to reduced time to recurrence (*P* = 0.09) (data not shown).

As both markers showed a tendency to impact in the time to recurrence, a combination of these 2 proteins was analyzed. For this purpose, the patients were grouped as high-risk (ATF6α^high^p-p38^low^) and low-risk (remaining combinations). The combination of ATF6α^high^ and p-p38^low^ was strongly associated with a higher risk of recurrence (HR 2.918, 95% CI 1.259–6.761, *P* = 0.013). Survival curve showed statistically significant differences for time to recurrence between these 2 groups of patients (*P* = 0.008; Fig. 1B). The median time to recurrence for the patients expressing ATF6α^high^p-p38^low^ was 8 months (range 3–13) compared with 21 months (range 6–36) for the low-risk group.

In the univariate analysis of clinical variables, only tumor grade (HR 3.256, 95% CI 1.283–8.266, *P* = 0.013) remained significantly associated with time to recurrence in conjunction with ATF6α^high^p-p38^low^ signature (HR 2.918, 95% CI 1.259–6.761, *P* = 0.013) (Table 2). Therefore, tumor grade was the only covariate used for adjustment. After multivariate Cox regression analysis, the combination of ATF6α^high^ and p-p38^low^ (HR 2.705, 95% CI 1.148–6.376, *P* = 0.023) and the tumor grade (HR 2.886, 95% CI 1.113–7.484, *P* = 0.029) remained significant (Table 2).

**TABLE 2:**
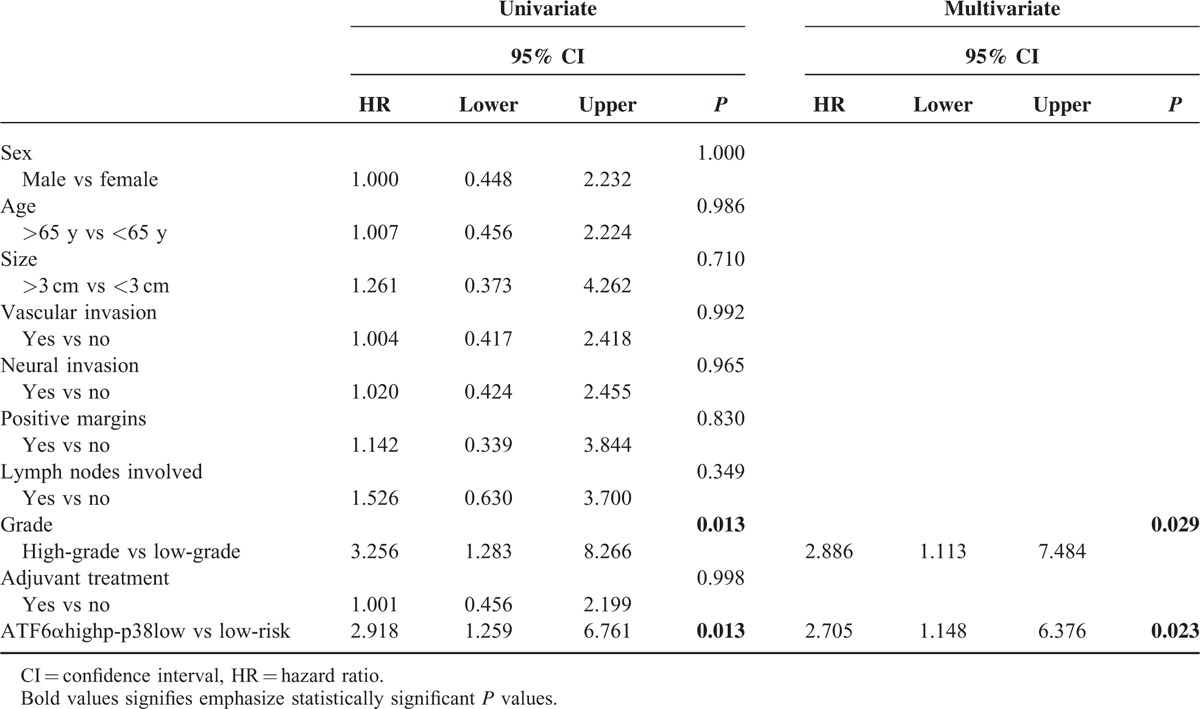
Univariate and Multivariate Analysis for Time to Recurrence

Furthermore, we performed Kaplan–Meier analysis for overall survival. The correlation of patients expressing ATF6α^high^ and p-p38^low^ with lower overall survival was close to be significant (*P* = 0.062, Fig. 1C). Median overall survival of patients expressing ATF6α^high^ and p-p38^low^ was 15 months (range 6–23), although low-risk patients did not reach the median value. Although ATF6α^high^ and p-p38^low^ did not achieve significance in overall survival analysis, this result supports the role of this protein signature as a high-risk factor for these patients.

These results suggested that the combined analysis of ATF6α and p-p38 could be used as a potential biomarker for high risk of recurrence in resected biliopancreatic adenocarcinoma patients.

## DISCUSSION

Up to date, biliopancreatic cancer is one of the most aggressive solid neoplasms, and incidence is rising worldwide. Prognosis of this cancer is still determined by the histopathological grading,^34,35^ and surgical resection is the best option so far to improve survival.^36^ Therefore, adjuvant therapy, usually based on gemcitabine, has a scarce benefit in survival, being mostly used as a palliative intent.^26,37^ Indeed, adjuvant therapy is administered after surgical resection without any clinical consensus and depends on oncologist know-how advised by a multidisciplinary team of experts. The current US FDA-approved marker for biliopancreatic carcinoma, CA19-9, is not recommended for its use in disease recurrence nor for response to therapy prediction.^27^ Thus, the decision whether or not to treat these patients and the type of adjuvant chemotherapy is always compromising for an oncologist. For this reason, medical community demands biomarkers to improve and personalize the treatment.^28^

ATF6α is a factor of ER stress response considered a biomarker involved in multiple pathways, such as survival and angiogenesis,^27^ and it has been related to high-grade transformation^29,32^ and chemotherapy resistance.^31,32^ Furthermore, ATF6α knockdown induced apoptosis of pancreatic cells after p38 phosphorylation,^20^ so ATF6α seems to be crucial for pancreatic cell survival maintenance. Independently, it has been described that overexpression of p-p38 in biliopancreatic cancer patients significantly improved median overall survival compared with those with low expression.^18^ Moreover, in patients who had completed adjuvant therapy, median overall survival was significantly higher for patients overexpressing p-p38.^18^

In this study, survival analysis was performed to analyze the correlation of ATF6α and p-p38 expression with outcome of the patients. Expression of both markers showed a trend to be related to the time to recurrence. We consider that significance was not reached due to the limited sample size. For this reason, we combined both markers to improve the predictive ability of our model. The 2-marker prognostic signature, based on ATF6α^high^ and p-p38^low^ levels, showed highly significant association with time to recurrence (*P* = 0.008) and a high trend with overall survival (*P* = 0.062). Cox hazard model showed not only ATF6α^high^ and p-p38^low^ signature (HR 2.705, *P* = 0.023) associated with poor outcome but also tumor grade (HR 2.886, *P* = 0.029).

To the best of our knowledge, adjuvant chemotherapy is an important factor to indicate early recurrence. Statistical analysis revealed no significant differences between treated and untreated arms. This result might be expected since there is no proof of any advantage of adjuvant or additive chemoradiation.^34,38^ Concerning positive margins and lymph node involvement, they did not achieve significance in our study and they should normally be significantly associated to poor outcome; however, this is not always the case. Neither positive margins nor lymph node involvement remained significant for time to progression and overall survival analysis in a phase II clinical trial which enrolled 48 resectable biliopancreatic cancer patients.^35^ Moreover, Andren-Sandberg^39^ classed lymph node positivity as a controversial variable for predicting survival.

**FIGURE 2:**
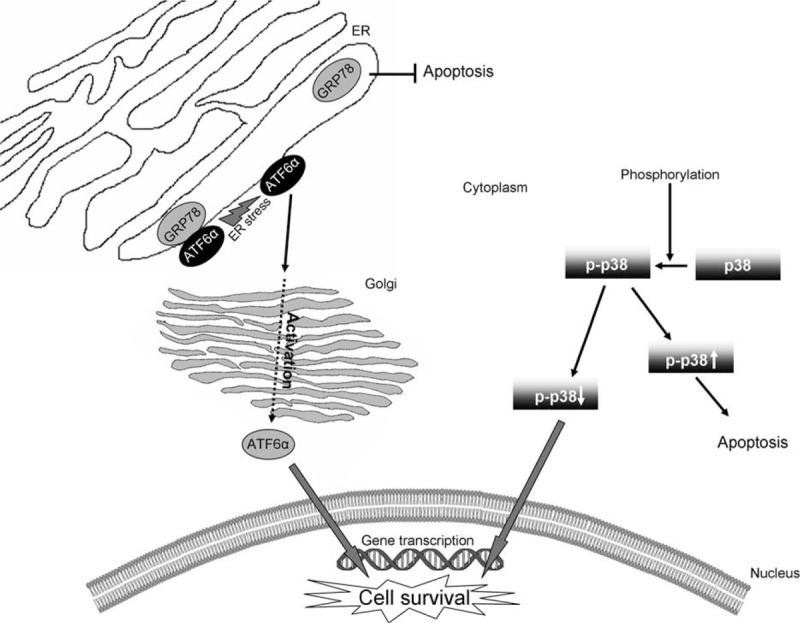
Proposed model by which ATF6α and p-p38 could modulate pancreatic cancer survival. ER = endoplasmic reticulum.

These results suggest the combination of ATF6α^high^ and p-p38^low^ as a novel marker associated with poor outcome in resected biliopancreatic cancer patients. We propose a model in which the high level of ATF6α and low levels of p-p38 could influence directly over cell survival (Fig. 2). In normal conditions, ATF6α is linked to glucose-regulating protein 78 (GRP78), but in ER stress, which is critical for tumor cells, both proteins dissociate. GRP78 prevents tumor cells from apoptosis, and ATF6α translocates to Golgi where it is processed and migrates to the nucleus where it stimulates transcription of survival genes.^25^ On the contrary, p38 is activated by phosphorylation through mitogen-activated protein kinase (MAPK) signaling pathway, then p-p38 translocates to the nucleus. Whereas high levels of p-p38 induce cell apoptosis, low levels of p-p38 promote cell survival.^40^ The joint effect of both proteins on cell survival would impact the outcome of biliopancreatic cancer patients.
